# Genealogy-Based Methods for Inference of Historical Recombination and Gene Flow and Their Application in *Saccharomyces cerevisiae*

**DOI:** 10.1371/journal.pone.0046947

**Published:** 2012-11-30

**Authors:** Paul A. Jenkins, Yun S. Song, Rachel B. Brem

**Affiliations:** 1 Computer Science Division, University of California, Berkeley, California, United States of America; 2 Department of Statistics, University of California, Berkeley, California, United States of America; 3 Department of Molecular and Cell Biology, University of California, Berkeley, California, United States of America; University of Cambridge, United Kingdom

## Abstract

Genetic exchange between isolated populations, or introgression between species, serves as a key source of novel genetic material on which natural selection can act. While detecting historical gene flow from DNA sequence data is of much interest, many existing methods can be limited by requirements for deep population genomic sampling. In this paper, we develop a scalable genealogy-based method to detect candidate signatures of gene flow into a given population when the source of the alleles is unknown. Our method does not require sequenced samples from the source population, provided that the alleles have not reached fixation in the sampled recipient population. The method utilizes recent advances in algorithms for the efficient reconstruction of ancestral recombination graphs, which encode genealogical histories of DNA sequence data at each site, and is capable of detecting the signatures of gene flow whose footprints are of length up to single genes. Further, we employ a theoretical framework based on coalescent theory to test for statistical significance of certain recombination patterns consistent with gene flow from divergent sources. Implementing these methods for application to whole-genome sequences of environmental yeast isolates, we illustrate the power of our approach to highlight loci with unusual recombination histories. By developing innovative theory and methods to analyze signatures of gene flow from population sequence data, our work establishes a foundation for the continued study of introgression and its evolutionary relevance.

## Introduction

One of the most fundamental questions in population genetics is how organisms acquire the novel alleles that serve as raw material for evolutionary innovation. Ancestral and de novo mutations, and their shuffling by recombination, provide much of the genetic variation in a given population. Gene flow between isolated populations within species, or introgression between species, can serve as an additional source of novel alleles when reproductive isolation is not complete. Reports in many taxa have identified introgressed haplotypes that have conferred an evolutionary advantage in the recipient population [1]–[4]. As such, detecting signatures of introgression has become a primary tool in the search to understand how organisms innovate during adaptation to new environments.

Powerful genomic analyses of gene flow have been developed for ecological model systems involving a known pair of populations in reproductive contact. These approaches seek to detect stretches of shared alleles between populations against a backdrop of otherwise high divergence, using broad and deep population genomic data sets that can be analyzed with metrics of population differentiation such as 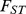 [5], as well as patterns of linkage disequilibrium [6]. However, in organismal systems where population structure is not well understood, the sources of acquired alleles in a given population may often be unknown or shallowly sampled in available sequence data. In this regime, detecting gene flow by many existing methods remains a challenge. In an alternative approach, studies of horizontal gene transfer routinely use phylogenetic methods to infer evidence of historical genetic exchange between species [7]–[10]. To date, the computational complexity of ancestral inference incorporating recombination has limited the use of genealogy-based analyses within species, and, as a consequence of such methodological limitations, the extent and evolutionary relevance of gene flow between divergent populations is largely unknown in many systems.

In this paper, we propose a scalable genealogy-based method to detect candidate cases of alleles that are acquired by a population through gene flow from unsampled sources and are maintained to the present. Specifically, our aim is to develop an efficient approach that would 1) enable genome-scale analyses of candidate cases of haplotypes acquired by gene flow events between divergent populations, 2) operate on shallowly sampled population genomic data, 3) allow fine-scale inference of the boundary positions of those genomic regions with alleles putatively acquired by gene flow, and 4) provide signatures of gene flow which could be analyzed in a theoretical framework.

The key fact that we exploit in our work is that boundaries of genomic regions inserted by gene flow correspond to recombination breakpoints. This holds even when the source population is not sampled; the boundaries of such a region are still detectable provided we sample *some* population in which the inserted haplotype is not fixed. Hence, our method is applicable to detecting the acquisition of haplotypes such that complete fixation has not yet occurred—which could occur if, for example, there is structuring within or between populations from which the samples were taken. Our goal is to infer historical recombination events from sampled data, and then consider a certain subset of recombination events to test for signatures of gene flow events between divergent populations. We note that there may be no qualitative difference between the signal from an inter-specific introgression event and that of a transient gene flow event from a strongly divergent strain of the same species, and our methods do not distinguish the two. Throughout, where we refer to “introgression” events, we do not reserve the use of this term for inter-specific gene flow.

**Figure 1:**
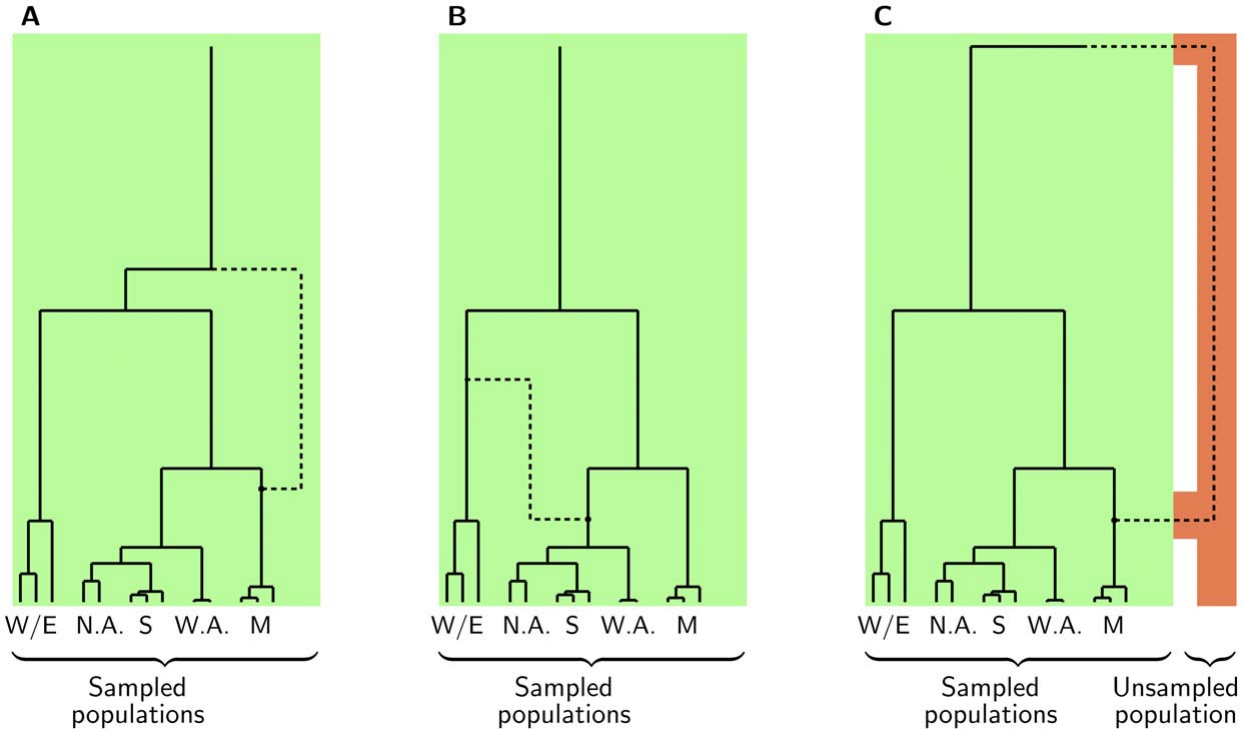
Examples of recombination events that create an outgroup clade. Each panel shows an example of a local genealogical tree (solid lines) for the currently sampled yeast populations (green), with samples from the wine/European (W/E), North American (N.A.), sake (S), West African (W.A.), and Malaysian (M) populations. In each panel, a dot indicates a recombination event. The dashed line indicates the recombinant lineage, which determines a new local tree on the other side of the breakpoint in which the three Malaysian strains are now at the outgroup position. In the absence of gene flow from an unsampled donor, two possible causes are **A**, the recombination occurred in a lineage ancestral to the Malaysian samples; and **B**, the recombination occurred in a lineage not ancestral to the Malaysian samples. **C**, Gene flow into the Malaysian population from an unsampled source (orange) will manifest as an inference of the former type of recombination.

In this paper, we say that a population forms an *outgroup* at a given site if one of the two branches adjacent to the root in the genealogy at that site subtends all samples from that population and no samples from other populations. An example is illustrated in the gene genealogy of yeast populations in Figure 1 A: the solid lines determine a genealogy in which the clade of yeasts of wine/European origin forms an outgroup. In what follows, as we use gene genealogies to investigate signals of gene flow between divergent populations, we focus on the extreme case where the ancestral lineage of a population undergoes recombination and modifies the population's outgroup status; that is, on one side of the associated recombination breakpoint, the population forms an outgroup, while on the other side of the breakpoint, it does not. Our strategy is motivated by the previous observation [8] that genetic exchange between a given genome and another, as yet unsampled, donor population divergent from the rest of the sample tends to place the recipient adjacent to the root of a genealogical tree. This idea is summarized in Figure 1. In the absence of gene flow from unsampled sources, we expect recombinations to occur both in the lineage of the population becoming the outgroup (Figure 1 A) and in other lineages (Figure 1 B). If additionally there is gene flow from an unsampled source, we expect to see an excess of the former type of recombination event (Figure 1 C).

As a testbed for our method, we analyze the recently reported genomic sequences of environmental isolates of budding yeast [11]. Well-defined phylogenetic populations have been identified in this species, but the prevalence of genetic exchange between the populations is incompletely understood [11]–[14]. To infer historical recombination events, we employ an efficient algorithm to reconstruct, for each gene, a collection of ancestral recombination graphs (ARGs) that encode putative genealogical histories of the sample. We then employ a theoretical framework based on coalescent theory to test for statistical significance of the frequency of recombination events that modify a population's outgroup status, to evaluate the evidence for gene flow from unsampled sources. Implementing these methods for application to empirical yeast data, we illustrate the power of efficient genealogy-based methods to highlight candidate cases of gene flow between divergent populations.

## Materials and Methods

### Sequence data

We downloaded from [11] whole-genome alignments of environmental yeast isolates and scripts to extract sequences. The prevalence of missing alleles in the original raw sequence data for these genomes rendered ancestral reconstruction intractable for the latter sequences, owing to computational constraints. Instead, we used yeast genomes that had undergone error correction by a genealogy-based method [11]; we did not investigate any potential bias from such corrections in our genealogy inferences, but we reasoned that a likely advantage of the correction strategy would be a reduction in artifacts arising from sequencing errors. The available data comprised the genomes of 21 yeast isolates: three from each of the sake and Malaysian populations, two from each of the North American and West African populations, and 11 from the wine/European population. To improve efficiency and avoid sample size effects, and to focus on inter-population exchanges, we randomly subsampled three wine/European strains (DBVPG1373, L-1374, and YJM978) for use in our analysis. We divided the data into a window for each gene defined by the open reading frame (ORF) plus one kilobase up- and downstream, or as far as the next ORF, whichever was smaller. The reference sequence for *S. paradoxus* was used to infer the ancestral allele at each site. To obtain an incidence matrix of single nucleotide polymorphism (SNP) data, we scanned each dataset for sites exhibiting variation in our sample. Unaligned regions or structural variants (labeled ‘ = ’ in the data) were excluded; indels were treated in the same way as SNPs. Those sites for which *S. paradoxus* could not resolve the ancestral allele unambiguously (such as triallelic sites) were excluded. We note that despite this preprocessing, we still expect our results to be affected to some degree by sequencing errors, recurrent mutations, and errors in assuming that *S. paradoxus* carries the ancestral allele at every site polymorphic in *S. cerevisiae*.

### Genealogical reconstruction

For each of 5842 yeast genes, we inferred a collection of explicit genealogical histories, or ancestral recombination graphs (ARGs), examples of which are given in Dataset S1. To balance the needs of efficiency and accuracy, we reconstructed ARGs using ideas based on parsimony. We used the software kwarg [15], which reconstructs evolutionary histories with a minimal or near-minimal number of recombination events under the infinite-sites assumption. This is in a manner similar in spirit to the Margarita software package [16] which has been applied to yeast data previously [11]. While Margarita is based on a heuristic algorithm, kwarg is closely based on an *exact* method for reconstructing ARGs with a minimal number of recombination events [15]. Further details of its implementation and the software parameters we used are provided in Text S1. We reconstructed 100 ARGs for each gene in the genome. For genes with complex histories, these ARGs would sometimes exhibit variability in properties such as the total number of inferred recombination events. In analyzing recombination scores as described in the subsequent sections, we took the mean score across this set of inferred ARGs for each region; in analyzing the identity of the outgroup clade for the genealogy of a given region, we took a majority vote across the inferred ARGs. With our choice of parameters, the running time for each chromosome on a single 3 GHz Intel Xeon processor was roughly two weeks.

### Recombination score

Using our inferred ARGs, we first counted the *number* of recombination events occurring to ancestors of samples from each yeast population. A recombination event can occur in a lineage which is ancestral to some or all samples from a given population, or to some or all samples from the other populations. In order to assign an inferred recombination event as ancestral to a particular population, we defined a score to be attributed to each population, as follows. If we are counting recombination events occurring in ancestors to population 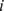 and a particular recombination event occurs in a lineage ancestral to a fraction 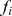 of the samples from 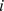, then this recombination event contributes a score of 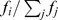 to population 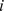, where 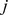 sums over all populations. For example, by this measure the score for the Malaysian population in Figure 1 A is 1, and the score for each of the North American, sake, and West African populations in Figure 1 B is 1/3. Other populations are assigned a score of 0.

**Figure 2:**
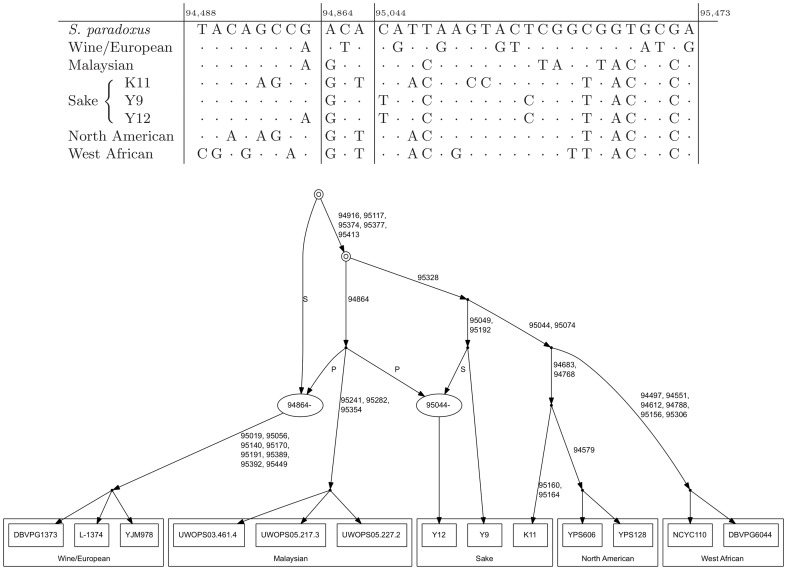
An example ancestral recombination graph for variation at the yeast gene encoding SAW1 (YAL027W) and flanking sequence. The upper table shows allelic variation across the seven distinct haplotypes in this region; dots represent sites identical to *S. paradoxus*. Vertical lines mark boundaries of the region and also two inferred recombination events in an inferred ARG, shown in the bottom panel. In the latter, nodes represent inferred coalescent and recombination events, and edges represent passage through evolutionary time. Labels on each edge represent the coordinates of sites that underwent inferred mutations along the respective branch. Extant *S. cerevisiae* genomes are at the leaves of the graph. Recombination vertices are indicated by the coordinate of the breakpoint and are shown as ellipses. At recombination vertices, edges labeled P indicate sites upstream of and including the breakpoint inherited from the “prefix” lineage, and edges labeled S represent downstream sites inherited from the “suffix” lineage. Annuli indicate vertices which are the most recent common ancestor of the whole sample at some site. Genomic coordinates for this region encompassed the open reading frame (ORF) and 200 bp upstream. The image is adapted from the output of kwarg, using Graphviz [37].

**Figure 3:**
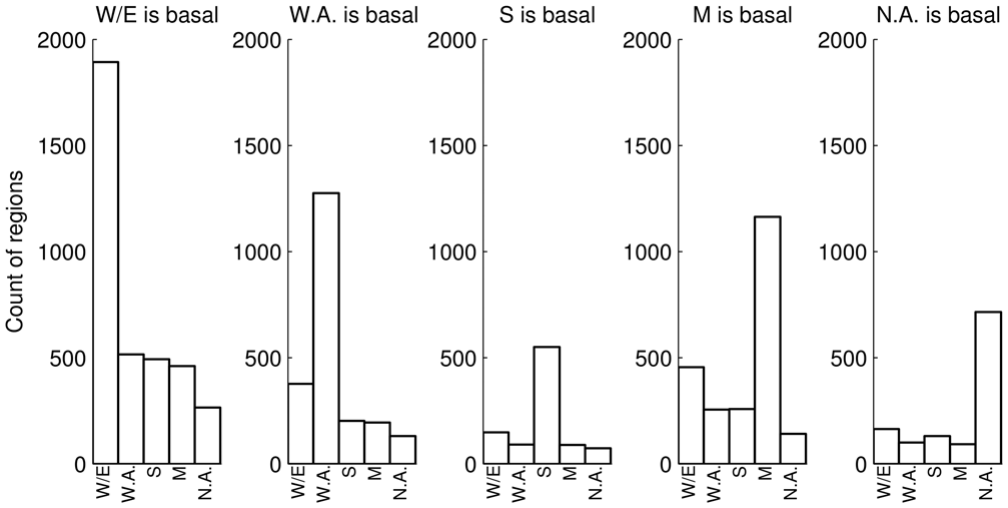
Inferred recombination by the outgroup clade at recombination block boundaries, in ancestral recombination graphs inferred from yeast sequence. Each panel represents results from regions of yeast genes in which the population indicated at the top occupied an outgroup position in the inferred genealogy. For each such region, the 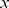-axis reports the identity of the population with the strongest evidence for disrupting recombination at the boundaries of the region, using the recombination score described in Materials & Methods. The 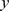-axis indicates the number of regions with inferred recombinations involving the respective population from the 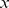-axis label. Populations are wine/European (W/E), North American (N.A.), sake (S), West African (W.A.), and Malaysian (M).

In many of our analyses, we focused only on recombination events demarcating regions for which a particular population was identified as an outgroup in the genealogy. We defined a population as being an outgroup at a site if one of the two branches adjacent to the root in the genealogy at that site subtended all samples from that population and no samples from other populations. For example, the wine/European population is an outgroup downstream of position 94864 in Figure 2; upstream of this position, no population is an outgroup. Over each interval for which a particular population was determined as an outgroup, we identified up to two *disrupting* recombination events at its boundaries—events which caused the necessary changes in tree topology to make this population an outgroup and which were identifiable inside the gene window. To report the strength of the evidence that the historical recombinations at the boundaries of the region involved population 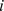, we computed recombination scores for 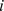 at each boundary as described above and summed the two. These recombination scores were used in Figure 3, which reports the identity of the population with the highest score. Genes for which independent ancestral reconstructions from kwarg did not agree on the number of recombination events were excluded from these analyses.

### Coalescent model

Our basic observation is that we expect (Figure 1 A, B) to see recombination events that change the status of a population so that it becomes an outgroup with respect to the other sampled populations, even in the absence of gene flow into the population from an unsampled source. However, should such gene flow occur, it causes an excess of recombination events in lineages ancestral to the population that becomes an outgroup (Figure 1 C). To evaluate empirical data from yeast ARGs, we therefore require a reproductive model to make predictions with respect to these different types of recombination event. As a first step, we sought a simple model which did not incorporate selection or gene flow from unsampled sources, and which could predict how often the recombination event that disrupted the outgroup status of a particular outgroup clade 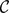 would occur in a lineage whose descendants are also members of 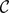. For this purpose, we first assumed a standard coalescent model, in which the whole population is mating randomly, population size is constant, no selection is acting, and so on. In this setting it was possible to make precise theoretical predictions, which we now develop in detail.

Intuitively, it is useful to think of an instance of transient gene flow, admixture, or introgression as changing the status of a clade from non-outgroup to outgroup between two local trees in the ARG (as is illustrated for the Malaysian strains in Figure 1 C). However, a mathematical presentation is simpler in the opposite direction: Consider the site at which a clade switches its status from outgroup to non-outgroup. Note that our concern is with the formation of outgroups with respect to the pooled collection of sampled populations, and our use of the term does not refer to a sample from some distant relative beyond the root of the genealogy. As we move across the site, we observe a ‘disrupting’ recombination event. Given the event, 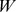, in which a particular set of 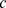 strains forms an outgroup clade, we seek the probabilities that a recombination event occurred at this site to toggle the outgroup status of the clade, and that it occurred either to an ancestor of members of this set (as in Figure 1 A, an event denoted 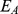) or to an ancestor of samples only from other populations (as in Figure 1 B, an event denoted 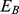). Our goal, therefore, is to compute the probabilities 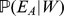 and 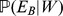, at least up to a common normalizing constant. (The probabilities do not sum to 1 because a recombination event need not be ‘disrupting’, nor even change the topology of the local tree [17].) Later, we use these to develop a formal statistical test for an observed excess of type 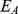 events relative to type 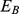, consistent with an additional contribution due to gene flow from unsampled sources.

**Figure 4:**
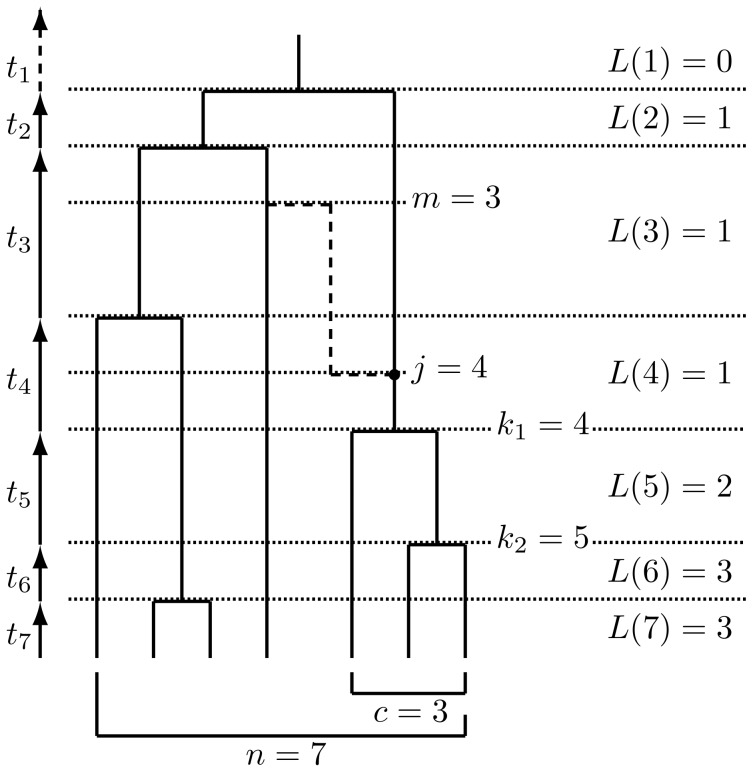
A coalescent tree for 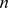
 **strains in which a set of** 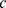 **strains form an outgroup clade.** A recombination occurs (marked by a dot), and the recombinant lineage (dashed line) recoalesces into the tree so that the clade is no longer an outgroup (satisfying 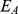). Notation is defined in the main text.

Since we consider the effect of the single recombination event, we are interested in a single, local, tree topology embedded in the ARG, and its neighboring tree on the other side of the recombination breakpoint. Denote the local tree topology in which the given set of strains is an outgroup by 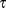, the duration of time in this tree for which there exist 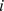 ancestors to the whole sample by 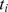, the number of ancestors of the sample when the recombination event occurred by 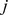, and the number of remaining ancestors of the sample when the new recombinant lineage coalesced back into the tree by 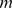. This notation is illustrated in Figure 4. We refer to the number of ancestors of the whole sample at a given time back as the *level*. Let 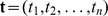 denote the duration of each level. We first consider the probability 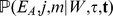, which is given by the product of the probabilities that

(a) a recombination event occurred,
(b) it occurred at level 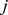,
(c) the recombination event occurred to an ancestor of strains that are members of the outgroup clade,
(d) the recombinant lineage did not recoalesce for the rest of level 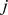 (if 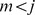),
(e) the recombinant lineage did not recoalesce during levels 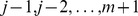,
(f) the recombinant lineage recoalesced at level 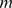,
(g) the recoalescence occurred with a lineage *not* ancestral to the outgroup clade.

Let 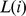 be the number of lineages ancestral to the outgroup clade at level 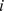, and 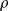 be the population-scaled recombination rate. Then the product of these probabilities is
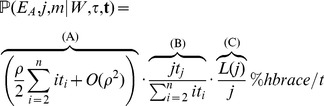


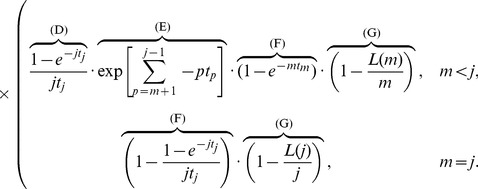
(1)In the above equation we ignore the probability that more than one recombination event occurred at this position, which occurs with probability 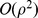. Equation (1) is valid for values of 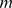 and 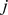 such that 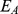 can occur:
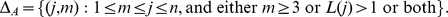
Note that not all plausible pairs of 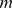 and 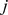 work; we must have 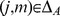. For example, if the recombination occurred at a level with 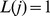, so the lineage undergoing recombination is ancestral to all members of the outgroup clade, and the recoalescence occurs at level 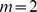, then this clade is still an outgroup in the new topology across the recombination breakpoint.

Equation (1) still applies for 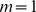 provided we define 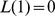 and 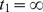. When 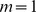, the recoalescence occurs further back in time than the most recent common ancestor (MRCA) of 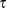. Under the standard coalescent model, 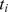 is a realization of an 
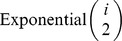
 random variable, for each 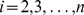, and so, summing (1) over 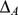 and integrating over the distribution of 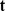, we obtain:
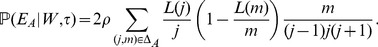
(2)In a similar manner, we find:
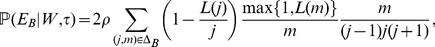
(3)where
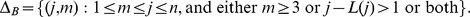
Finally, we need to integrate over the distribution of 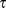, all possible tree topologies in which a given set of strains forms an outgroup clade. Equations (2) and (3) depend on 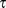 only through 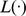. This dependence is encapsulated by noting that, if 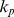 is the total number of lineages remaining after the coalescent event that reduces the size of the outgroup clade to 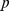, then for 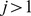 we have 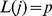 where 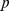 satisfies 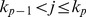. So for a clade of size 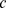, we must have that 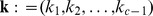 lies in
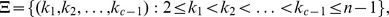
(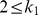 being necessary to ensure the clade of interest is an outgroup), and we may write
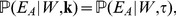
(4)where 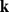 is consistent with 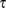. For example, for 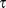 as in Figure 4, 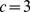, 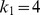, and 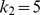. Thus, decomposing the probability of interest as
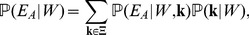
(5)it remains to find 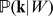. We now argue that this is uniform on 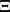. Observe that
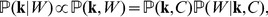
(6)where 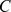 denotes the event that 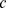 particular samples form a clade in the genealogy (but not necessarily an outgroup). Lineages ancestral to the 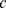 samples initially coalesce at rate 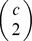, and the remaining lineages coalesce at rate 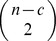. For a randomly drawn coalescent tree it is straightforward to show that
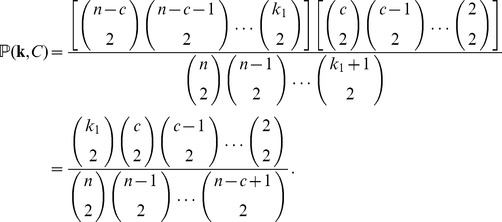
(7)In total there are 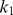 lineages remaining at the time the 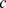 strains coalesce into a single lineage, cementing their status as a clade. With probability
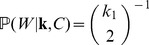
(8)the single lineage ancestral to this clade participates only in the final coalescence of the whole genealogy, in which case the clade is also an outgroup, satisfying 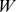 as well as 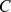. Thus, combining equations (6)–(8), we see that 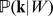 is independent of 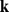, and we can bring 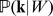 outside the sum in (5). Combining this observation with (2) and (4), we find:
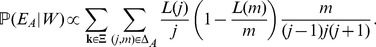
(9)In a similar manner,
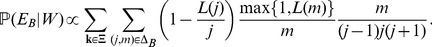
(10)The normalizing constants in (9) and (10) are the same, and so from these equations it is straightforward to compute the relative probabilities of the two events on 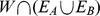, which was our aim. Specifically, we can find
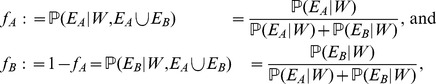
(11)which govern our expectations regarding the relative frequencies of the two types of recombinations events 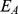 and 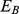 (Figure 1 A, B, respectively). The quantity 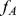 is used in Tables 1 and 2, below, and compared with empirical data in Tables 3 and 4. Because the lineage ancestral to all members of the outgroup clade persists further back in time conditional on the fact that the clade *is* an outgroup, 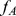 is substantial for most values of 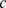 and 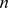, as noted below. There is in fact also an opposing effect, in which the strains of any given clade have a *younger* MRCA conditional on the fact that they form a clade. This provides less opportunity for genetic exchange with other strains, but manifested only for very small values of 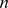 (Table 1).

**Table 1:** Recombination by the outgroup clade at block boundaries, in theoretical predictions from a simple coalescent model of a single panmictic population.

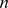	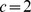	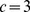
3	0.750	-
4	0.500	0.733
5	0.436	0.564
6	0.407	0.500
7	0.390	0.466
8	0.378	0.444
9	0.370	0.428
10	0.364	0.417
11	0.359	0.408
12	0.356	0.401
13	0.352	0.395
14	0.350	0.390
15	0.347	0.386
20	0.339	0.371
100	0.315	0.327

Each row represents results from a hypothetical genomic region in which a given set of 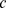 sequences forms an outgroup clade in the genealogy, in a sample of total size 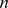. Listed is the probability that, under a panmictic coalescent model, the disrupting recombination event at the boundary of the region toggling the status of the outgroup clade occurs in a lineage ancestral to members of that same clade.

**Table 2:** Fractions of disrupting recombination events that occur in lineages ancestral to members of the outgroup clade in yeast ARGs, from simple coalescent simulations.

	Sample size	
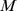	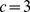	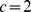
0.1	0.262	0.260
1	0.316	0.307
10	0.367	0.338
100	0.390	0.350
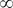	0.395	0.352

Each row represents results from a simulated genomic region in which a given set of 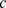 sequences forms an outgroup clade in the genealogy. Shown is the probability that a recombination event at the boundary of the region occurs in a lineage ancestral to members of the outgroup clade, when the outgroup clade has size 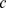 and the total sample size is 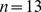. We assumed a structured coalescent model with five islands of equal size, allowing random mating within islands and symmetric migration between islands, governed by the population migration parameter 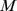. Probabilities were estimated by importance sampling, except for the model 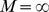 which was calculated exactly.

**Table 3:** Observed fractions of disrupting recombination events that occur in lineages ancestral to members of the outgroup clade in yeast ARGs.

Outgroup	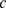	Fraction
Wine/European	3	0.366
Sake	3	0.398
Malaysian	3	0.378
West African	2	0.439
North American	2	0.442

Each row reports the results of analysis of ARGs inferred from empirical yeast data. Each fractional value indicates the frequency with which, given a region whose inferred genealogy positioned the indicated clade as an outgroup, the recombination event at the region's boundary occurred in a lineage ancestral to the outgroup clade. Total tree size was 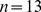 in each case. 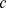, clade size.

**Table 4:** Evaluating observed fractions of disrupting recombination events that occur in lineages ancestral to members of the outgroup clade in yeast ARGs.

	Outgroup				
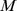	Wine/European	Sake	Malaysian	North African	North American
0.1	2.00 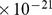	5.08 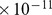	7.73 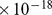	4.57 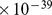	1.33 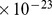
1	1.64 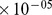	2.26 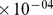	1.67 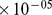	1.25 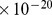	8.29 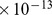
10	1	3.90 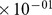	1	3.06 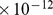	7.64 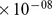
100	1	1	1	1.16 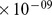	2.65 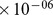
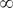	1	1	1	3.04 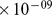	4.74 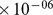

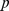
-values reporting significance of observations in Table 3 by comparison with model expectations in Table 2. Each row represents a comparison of inferred genealogies from yeast genome sequence with coalescent simulations under a model of isolation with migration, with the indicated population migration parameter 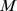. Values report one-tailed 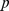-values for the likelihood of the observed data under the model, calculated according to binomial sampling of recombination events, and Bonferroni-corrected to account for the testing of five populations.

### Migration model

We generalized the results (9) and (10) to incorporate population structure by simulation. Specifically, we considered a simple island model in which each of the five yeast populations was isolated and was mating randomly within each population. In the basic simulations used to interpret much of the results below, each island was assumed to have the same effective population size 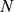; migration occurred symmetrically between islands and at the same rate, governed by the population migration parameter 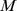. (On a coalescent timescale, the total rate of migration out of each island is 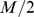.) This island model has been used previously to investigate gene flow in *S. cerevisiae* [13]. We simulated local tree topologies under this model, retaining for analysis only those for which a particular pre-chosen population was an outgroup clade. For an accepted tree 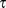, a set of times between coalescent and migration events was then simulated. A recombination event was placed uniformly at random on the branches of the tree, and the new recombinant lineage was allowed to migrate and to recoalesce with the tree at the prior rate specified for the simulation. To correct for the fact that under this proposal distribution each accepted tree experienced a recombination event with probability 1, each accepted tree was given an importance weight equal to its total branch length, 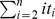. For this tree we recorded whether 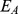 was satisfied, or 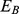, or neither. The latter outcome occurred when the coalescence of the recombinant lineage resulted in no disruption of the status of the outgroup clade, in which case the simulation was discarded. The fractions of the first two events across a large number (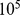) of accepted simulations provided an estimate of the relative probabilities of 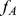 and 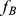 under this model [equation (11)]. To summarize, these methods enabled us to compute, for each choice of 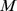, the expected fraction of disrupting recombination events that occurred in a lineage ancestral to the outgroup clade, 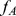, either exactly [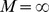, equations (9) and (10)] or by simulation [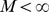, by importance sampling]. That our simulation results approach theoretical predictions as 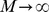 (Table 2) provides strong validation of the two methods.

We further estimated 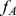 by simulation in the manner described above, but under a broader class of models, exploring the impact of perturbations from the symmetric island model (Table S2). First, we considered a model with more recent population substructure, in which each island appears from a previously panmictic population 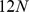 generations ago. We then further considered the effect of unequal effective population sizes, by estimating 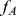 when the population A with the outgroup clade has either a five-fold larger or five-fold smaller effective population size, or when one other population B has either a five-fold larger or smaller effective population size. Similarly, we considered the effect of asymmetric migration by estimating 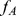 under a model in which the migration rate into population A was five, 10, or 50 times every other migration rate, or when the migration rate out of population A was five, 10, or 50 times every other migration rate. Finally, we considered various combinations of different population ages by estimating 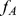 under a model in which islands are allowed to split from the ancestral panmictic population at different times. For example, population A can be modeled as older than the rest by maintaining all the other islands as fully panmictic until a more recent 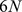 generations ago.

Realistic choices for 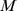 were estimated from the sequence data by calculating 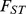 in each gene window. For a given pair of populations, 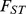 was estimated by one minus the ratio of within- to between-population diversity (eq. 3 in [18]), where diversity is given by the mean number of pairwise sequence differences. Each gene therefore provided an estimate of 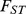 for each pair of populations. We then converted each point estimate of 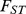 to a point estimate, 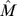, of 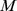 (eq. 7 in [18]). This collection of estimates also provided a measure of confidence in our estimation: we used this distribution, weighted by gene length and corrected for sites with missing data, to compute percentiles of our distribution of 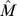.

### Model-based tests of the frequency of disrupting recombinations in the ancestry of the outgroup clade

We used predictions from our coalescent models and simulations to evaluate the genome-wide distribution of recombination events in empirical ARGs inferred from yeast data, as follows. For each genomic region in which a particular population formed an outgroup clade in our inferred ARGs, we tallied whether its disrupting recombination events occurred in a lineage ancestral to members of that same clade (event 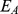). Under the null model above, and assuming the local tree at each disrupting recombination event was independent of every other tree, this tally follows a 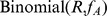 distribution, where 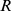 is the total number of disrupting recombination events inspected. We used one-tailed 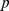-values from this distribution to test for an excess of such events. To account for the non-independence of recombination events, we conservatively scaled down 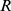 and our observation from the binomial distribution so that 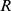 was equal to the number of *genes* rather than the total number of relevant disrupting recombination events (scaled by the fraction of intervals for which the given population was an outgroup).

Our predictions are based on the true genealogies being observed, but in reality the test will be based on *inferred* ARGs. Thus, both the random process of mutation and the parsimony underlying our ARG reconstruction algorithm could introduce additional sources of error and bias the test described above. To confirm that it is well calibrated despite such potential distortions, we applied it to a number of simulated datasets. For this purpose we simulated synthetic whole-genome datasets using ms [19]. We used the same sample sizes and number of gene windows as in the real dataset described under Sequence Data, matching each gene for length and number of polymorphic sites. A constant recombination parameter of 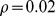 per bp was chosen to approximately match the number of inferred recombination events, using a coalescent scaling in terms of the population size of each deme, 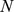 [19]. One such whole-genome dataset was simulated under each of two models: [(i)]

- A symmetric five-island model with migration parameter 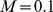 (i.e. the null model) as far back as 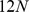 generations ago, beyond which all populations undergo fully random mating.
- A symmetric five-island model with 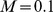 and an additional admixture event into the first of the five sampled populations from a sixth, unsampled population. The admixture was modeled as an instantaneous event 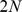 generations ago replacing 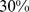 of the gamete pool of the recipient population. Aside from this sole admixture event, the sixth population is reproductively isolated. Farther back in time, 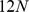 generation ago and beyond, all six populations are in contact again, with fully random mating.

We processed each of these datasets in the same manner as we processed the yeast dataset: namely, we carried out genealogical reconstruction using kwarg, calculated the relative frequencies of occurrence of events 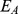 and 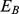 as described above, and computed one-tailed 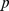-values based on the binomial distribution. Notice that carrying out this procedure on each dataset is highly computationally intensive, and it is not feasible to explore the effects of a wide range of models on the robustness of our test. Nonetheless, the control analysis on these simulated genomes should give an indication of whether the test is miscalibrated.

## Results

### Inference of historical recombination and gene flow

We set out to analyze the relatedness between environmental yeast isolates, using the whole-genome sequences of strains previously identified for their membership in five well-defined populations [11]: sake isolates from Japan, North American isolates from oak trees, West African brewing isolates, isolates from the Malaysian bertam palm, and isolates of European or vineyard origin. As described in Materials & Methods, for each of 5842 yeast genes, we inferred a collection of 100 ancestral recombination graphs (ARGs) using the software kwarg [15]. This approach reconstructs evolutionary histories with a minimal or near-minimal number of recombination events under the infinite-sites assumption. We were motivated to consider this approach because related methods have been used successfully in applications including fine mapping of disease loci [16], SNP detection and missing data imputation [11], [20]. Such a model-free, parsimony-based paradigm also avoids assumptions about the mating scheme in the ancestry of extant strains, and its efficiency enables the reconstruction of many plausible ARGs for every gene in the entire *S. cerevisiae* genome. Moreover, we note that parsimony-based methods [18], [21] have been well used in the past for estimating levels of gene flow from DNA sequence data. We interpret our sample of near-minimal ARGs, as reconstructed by kwarg, as an approximation of the true evolutionary history of each gene. Inference with kwarg provided ARGs for each of 5842 yeast genes (Figure 2 and Dataset S1 give examples), establishing for each gene the relationship between the strains of the five populations, using data polarized with respect to *S. paradoxus*. To avoid biases deriving from the more highly sampled wine/European population relative to the others in the data set, we subsampled three random wine/European strains for use in our analyses. We note that the windowing of the genome so that each gene is analyzed separately introduces an upper bound to the size of introgressed haplotypes that can be detected. Therefore, the distribution of lengths of putatively introgressed haplotypes as inferred by our method will not be representative of the true distribution. This windowing was made to ensure that analysis of the whole genome was computationally feasible, and we note that it would be straightforward to apply our method to larger contiguous regions of the genome.

We sought to use the yeast ARGs to trace patterns of ancient genetic exchange between yeast populations, focusing on loci harboring sequence signatures consistent with gene flow from unsampled sources. To this end, we first tabulated the number of recombination events inferred in ARGs of each gene and observed that most genes harbored few inferred recombinations, reflecting the parsimony of our inference approach (Figure S1). Next, we tabulated the identity of the outgroup clade in inferred ARGs. The results, summarized in Figure 3, revealed considerable variation in ARG tree topologies across loci as expected [11]. Using arguments similar to those given in equations (7) and (8) we reasoned that, if five lineages ancestral to each of the five populations were evolving according to a randomly mating population as we trace them farther back in time, then at any given locus, one of these lineages will be an outgroup with probability 0.1; with probability 0.5 none of these lineages will be an outgroup. In contrast to this expectation, 35.5% of loci supported an outgroup status in the tree for the wine/European population (Figure 3). We conclude that the model of a panmictic population is not sufficient to describe patterns of yeast genealogies, and that in particular, wine/European genomes are the most likely in the sample to display the outgroup status in a given ARG.

The simplest explanation for the outgroup positioning of a given clade 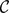 in an ARG does not invoke a model of historical genetic exchange. Rather, a population which has experienced ancient reproductive isolation, during which time gene flow with other populations was rare or absent, will be inferred as an outgroup to the rest of the species. We considered this notion a compelling interpretation for the excess of genomic regions whose inferred ARGs positioned the wine/European strains as an outgroup to the rest of the sample. To pursue analyses of gene flow more generally in yeast genealogies, however, we focused on two other models likely to explain most instances in which a given population 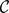 was inferred to occupy an outgroup position in an ARG, illustrated in Figure 1 for the case in which the set of Malaysian samples is the clade 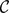 made an outgroup by a recombination event. As shown in Figure 1 C, under one model, 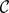 could acquire alleles by recombination from an unsampled donor population whose reproductive contact with the sampled population may be limited. On the other hand, alleles could be exchanged via recombination events involving the common ancestors of populations within the sampled metapopulation (Figure 1 A, B) —that is, between ancestors of the sampled populations for whom reproductive contact is ongoing. As shown by the dotted lines in Figure 1, the former model (Figure 1 C) must invoke recombination events in the ancestors of only the 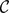 strains. The latter model (Figure 1 A, B) invokes recombination events in lineages ancestral to any population, provided they result in a change which drives 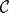 to the base of the local tree. For example, in Figure 1 B the recombination event results in a sharing of haplotypes among all of the wine/European, North American, sake, and West African lineages, putting the Malaysian clade as an outgroup.

To begin to evaluate these models of historical genetic exchange as interpretations of yeast ARGs, and ultimately to develop methods that identify candidate cases of gene flow into the sampled populations from unsampled sources, we examined the inferred instances of historical recombination at the boundaries of each locus where a given population was inferred to be an outgroup. For each such boundary position, we first identified the recombination event in the genealogy defining the breakpoint. We then developed a scoring scheme, described in Materials & Methods, to identify those populations subtended by the recombination. Figure 1 A shows an example in which a recombination event subtends the Malaysian isolates, and the example in Figure 1 B illustrates a case in which a recombination event subtends the North American, sake, and West African isolates. We tabulated the recombination scores for every stretch of the genome in which the inferred ARG placed a single population at an outgroup position. The results, shown in Figure 2, revealed a striking imbalance: most often, when a population occupied an outgroup position in the tree in a given genomic region, the recombination events at the boundaries of the region showed the strongest evidence for subtending that *same* population.

### Recombinations disrupting the outgroup status

To interpret the above finding, we focused on each recombination event that disrupted the status in the tree of strains of a given yeast population, changing their status from outgroup to non-outgroup as we scan along the sequence over the recombination breakpoint. Throughout, we refer to this type of recombination event as *disrupting*. We sought a theoretical estimate of the probability that a given disrupting event occurred in an ancestor of the outgroup, relative to the occurrence of such a recombination among the other lineages of the tree. We used the structured coalescent [22] to predict the prevalence of disrupting recombination events under a null model with no selection and no incoming gene flow from unsampled sources.

We first considered a simple model of a single, neutral, panmictic population to describe the entire yeast data set, which enabled us to obtain a closed-form expression for the probability under this model that a disrupting recombination event occurs in an ancestor of the outgroup strains (see Materials & Methods). As Table 1 shows, at a locus with a given population 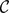 occupying an outgroup position in the tree, under the standard coalescent model the disrupting recombination event is generally more likely to involve an ancestor of 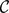 than to involve some other lineage, in a manner qualitatively consistent with our empirical findings from yeast ARGs. In the model, for example, if 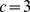 samples form an outgroup clade in a genealogy involving a total of 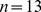 strains, the disrupting recombination event occurs in a lineage ancestral only to members of that clade with probability 0.395, much higher than a naïve guess such as 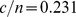 (Table 1). This shift manifests because the lineage of the most recent common ancestor of the members of the outgroup clade conditionally persists further back in time, maximizing the opportunity for genetic exchange between the strains of this clade and other individuals.

We also used a simulation strategy to investigate a null model more closely approximating yeast population structure [13]: a series of isolated and panmictic populations exchanging rare migrants. As shown in Table 2, in coalescent simulations under this model, disrupting recombination events again involved the outgroup clade more often than would be expected under a naïve random model (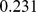; see above). Thus, theory and simulation of neutral models in the absence of gene flow from unsampled sources predicted that, for loci at which a particular clade occupies an outgroup position in the inferred tree, recombination events at the locus boundaries have often occurred in the lineage leading to that clade. Such a propensity is in qualitative agreement with the trends in the ARGs inferred from yeast sequence (Figure 3). We conclude that at many loci, the outgroup position of a set of yeast isolates in a genealogy is at least partially explained by inheritance through random coalescence events involving the currently sampled genomes. Conditioning on a given such outgroup position, we detect signatures of recombination at the boundaries of the locus owing to genetic exchange events ongoing throughout history at the flanking regions of this region.

Having established that panmictic and simple migration models could drive qualitative patterns of recombination at loci with genealogies displaying outgroups, we next asked whether a quantitative interpretation of the data would shed light on the prevalence of candidate cases of gene flow and introgression from unsampled sources in yeast evolution. In formulating a test for this purpose, we again considered boundaries of the yeast genomic regions where a given population 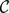 was inferred to be the outgroup clade, and we calculated, across this set, the frequency of recombination events involving the 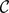 lineages relative to events involving all other lineages. We sought to evaluate this observed frequency against the analogous quantity from simulations of neutrally evolving, isolated populations whose exchange of migrants we modeled by the population migration parameter 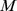. We first generated empirical estimates of 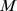 via metrics of the population differentiation parameter 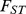 as follows. For each pair of populations and for each gene we used sequence diversity to estimate 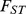, which in turn provided an estimate of 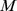 following the procedure in [18] (see Materials & Methods). The results, summarized in Table S1, show that data from every pair of populations were consistent in estimating 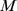 close to 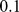, with 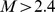 outside the estimate of 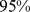 of genes in most calculations (Table S1).

Before using our estimated migration parameter to evaluate the frequency with which recombinations were inferred in lineages ancestral to outgroup clades in yeast ARGs, we developed a simulation-based check on the power and specificity of our approach. For this purpose, we simulated whole genomes as described in Materials & Methods, first under (i) a symmetric five-island model matching the real data for sample sizes, gene lengths, and levels of polymorphism, migration, and recombination; and second under (ii) a similar model with the sole addition of an admixture event into one of the five populations from an unsampled source. We then evaluated these simulated data against predictions from the coalescent model with migration. Results are shown in Tables 5 and 6. As expected, under model (i), observed frequencies of recombination events involving the lineage of the outgroup population were close to those predicted by the coalescent. Under model (ii), observations of the population undergoing incoming gene flow from an unsampled source deviated from the predictions of the coalescent, to a degree significant at the 5% level when the model was consonant with the true underlying migration regime; we observed no such deviations for the remaining populations, again as expected. We conclude that our test is indeed able to identify a population subject to gene flow from an unsampled source, under the reasonable model we consider here. We note that statistical power is diminished if the modeling step assumes that the true value of 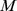 is large; thus, a conservative application of this test to real data would be to *overestimate* 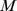.

**Table 5:** Fractions of disrupting recombination events that occur in lineages ancestral to members of the outgroup clade in ARGs, from complex coalescent simulations.

Outgroup	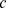	Model (i)	Model (ii)
# 1	3	0.295	0.366
# 2	3	0.244	0.222
# 3	3	0.219	0.204
# 4	2	0.245	0.243
# 5	2	0.213	0.189

Observed fractions of disrupting recombination events in lineages ancestral to members of the outgroup clade, from simulated genomic data for a hypothetical population with five populations (labeled #1–#5). Each row reports analyses of genomic regions in which the indicated population of sample size 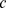 occupied an outgroup position in the inferred genealogy, where the total tree comprised 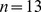 sequences in each case. Across all such regions was tabulated the frequency with which boundaries of the regions were defined by recombination events occurring in lineages ancestral to the *same* outgroup population. Genomic data was simulated under two models: (i) a symmetric five-island structured coalescent model with 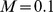, and (ii) a model in which again 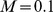 between the five populations, but also with an admixture event into population #1 from an otherwise isolated and unsampled sixth population. In both models the populations revert to a completely panmictic metapopulation beyond 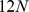 generations ago.

**Table 6:** Evaluating observed fractions of disrupting recombination events that occur in lineages ancestral to members of the outgroup clade in ARGs from complex coalescent simulations.

		Outgroup				
	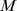	#1	#2	#3	#4	#5
Model (i)	0.1	6.19 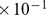	1	1	1	1
	1	1	1	1	1	1
	10	1	1	1	1	1
	100	1	1	1	1	1
	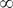	1	1	1	1	1
Model (ii)	0.1	1.20 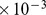	1	1	1	1
	1	3.31 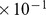	1	1	1	1
	10	1	1	1	1	1
	100	1	1	1	1	1
	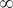	1	1	1	1	1

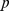
-values reporting significance of observations in Table 5 by comparison with expectations in Table 2. Calculation of 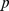-values and theoretical predictions are as described for Tables 2, 3, and 4.

With this evidence in hand that our coalescent-based test of recombination in lineages ancestral to outgroup clades performed as expected on simulated data, we applied the test to true inferences from yeast ARGs. Results, reported in Tables 2, 3, and 4, provided strong evidence in every yeast population for a departure from the assumed migration model with 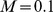. Remarkably, even under the conservative assumption of larger 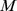 values (see above), neutral models of genetic exchange between the populations of the sample still could not fully account for the prevalence of recombination by outgroup lineages (Table 4). Conclusions from the wine/European, sake, and Malaysian clades were unchanged when we analyzed two strains from each rather than three, ruling out an influence of the number of isolates on the significance results in this test (data not shown). We conclude that over and above the predictions of simple population genetic models, yeast genealogies exhibit an excess of disrupting recombination events by outgroup clades.

To investigate the dependence of this conclusion on the assumptions of our symmetric island model, we explored by simulation the consequences of differences in demography between populations. Models in which migration rates or population ages were not identical across the five yeast clades had modest impact on the predicted frequency of disrupting recombination events ancestral to the outgroup clade (Table S2B, S2C), with the exception of positing unrealistically high migration rates between some populations, as discussed above. For models in which one yeast clade 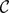 had a larger effective population size than the rest, the frequency of disrupting recombination events ancestral to the outgroup was inflated relative to the symmetric model when 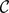 was the outgroup, and reduced otherwise (Table S2A). The latter finding renders it unlikely that the recombination patterns we observed in ARGs inferred from empirical yeast data are a consequence of increased size of any one population, since for most parameter sets, the excess of disrupting events in outgroup lineages in the empirical ARGs relative to symmetric models was not particular to any one clade (Tables 3, 4). We conclude that our simulation methods can be a powerful tool in the evaluation of recombination patterns in empirical ARGs, and that the frequency of disrupting recombination events in yeast ARGs exceeds the predictions of most parameter combinations we explore here.

## Discussion

Commonly used methods to detect introgression from DNA sequence data can require a depth and breadth of genome sampling that is unavailable for many populations of interest. Alternative strategies have the potential to revolutionize the study of migration and natural selection in non-model systems. We have developed analysis approaches based on genealogical inference to trace evidence for gene flow into outgroup lineages. We established a scoring scheme for genetic exchange events involving populations in genealogies, and we derived theoretical predictions, in conjunction with simulated data, to analyze this metric. In application to genome data from environmental yeast isolates, this approach revealed evidence for recombination events on outgroup lineages over and above the expectation from many neutral models of the population sample. The possibility that such recombination events may be a product of gene flow from unsampled sources is supported by the known impact of introgression on deviations from predictions of the standard coalescent [23], [24].

A key conclusion from our work is that patterns suggestive of gene flow from unsampled sources can be detected in many yeast populations. The notion of a sink of undiscovered sequence diversity in wild yeast would be consistent with the prevalence of singleton alleles in currently sampled yeast genomes [11]. Likewise, the notion of frequent genetic exchange with unsampled populations echoes extensive previous reports of evidence for recombination between sampled *S. cerevisiae* strains [11], [13], [14], [25], [26] and between *Saccharomyces* species [27]–[33]. The emerging picture is that reproductive contact between isolated yeasts is relatively common, likely as a function both of trafficking by humans and natural dispersal mechanisms, including insects [34].

Although we have focused on evidence for gene flow in this work, our inferred genealogies hold promise as a powerful complement to current approaches [35], [36] for analysis of larger-scale population genetic questions in the yeast system. Surprisingly, for example, in our ARGs the wine/European strains most often occupied the outgroup position of gene genealogies. This is in intriguing contrast to analysis of smaller data sets [12] and suggests a model in which the wine/European population underwent a period of ancient reproductive isolation, allowing the accumulation of distinct alleles that manifest as an outgroup position in inferred ARGs. Alternatively, the outgroup positioning of the wine/European strains in our ARGs could be the consequence of genetic exchange with divergent populations through recent, worldwide trafficking of vineyard strains. However, since our methods focus on ancient genetic exchange events inferred in the common lineage leading to the wine/European isolates, recent admixture events involving one or a few strains are unlikely to influence our results. As such, our findings are most consistent with the notion that the wine/European clade of *S. cerevisiae* represents an ancient and divergent population which in recent times has come into contact with the rest of the sample. This effect would likely be part of a complex and largely unknown history of genetic exchange across the full set of yeast isolates in the current sample [11], [13], [14], [25], [26], of which reproductive models described in this work must necessarily be only an approximation. Nevertheless, our results suggest that scalable genealogy-based methods, of the type we develop here, will serve as a springboard for future analysis of genetic exchange between these sampled populations, and of the effects of natural selection on exchanged material in their environmental niches.

## Supporting Information

Text S1 **Description of kwarg.**
(PDF)

Dataset S1 **Sample output of genealogical reconstructions using kwarg.** An ancestral recombination graph is provided for each gene in the *S. cerevisiae* genome, in dot format [37]. See Figure 2 for an example.
(TGZ)

Figure S1 **Distribution of the number of inferred recombinations across yeast genes.** Shown is the inferred minimum number of recombination events per gene from ancestral recombination graphs for 5842 yeast genes; genes with five or fewer SNPs and those for which independent ancestral reconstructions did not agree on the number of recombination events were eliminated from the data set.
(TIF)

Table S1
**Genome-wide estimates of the migration parameter** 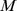 **under a symmetric island model, as inferred from** 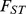 **.** Each row reports estimated population differentiation and migration parameter values for one pair of yeast populations. 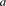 In a given gene window, 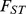 is calculated as one minus the ratio of within- to between-population diversity, as given by the mean number of pairwise sequence differences. Mean 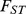 is the mean of this value across genes, weighted by length. 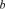 A point estimate of the migration parameter 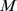 in a symmetric five-island model, inferred from mean 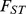 and using a theoretical prediction of the relationship between 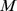 and 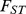 (see Materials & Methods). Also given are percentiles for this estimate, inferred from the distribution of 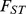 across genes.(PDF)

Table S2
**Changes in** 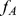 **caused by deviations from the symmetric five-island model.** Each cell reports an estimate, by simulation, of 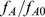, where 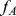 is the fraction of disrupting recombination events toggling the outgroup status of population A which also occur in a lineage ancestral to population A, under the indicated model; 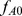 is the analogous quantity for a five-island population model with constant, symmetric migration with parameter 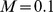 and reference effective population size 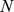 per population, which reverts to a fully panmictic population 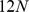 generations ago. Under this model, 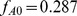. **A**. One population has a different effective population size. The first column perturbs the population size of the population experiencing the disrupting recombination event, denoted A, while the second column perturbs the population size of one of the other four populations, denoted B. **B**. The population whose outgroup status is disrupted, A, experiences higher incoming (first column) or outgoing (second column) migration rates, relative to the other populations. **C**. An alternative model in which certain populations have an older origin. In the first column, each island joins a single, panmictic, population 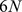 generations ago, with the exception of population A, which joins the panmictic population at the time indicated. In the second column, it is instead one of the other four populations, which we call B, that joins the ancestral panmictic population farther back in time.(PDF)

Table S3
**Parameters for parsimony score used in kwarg to reconstruct ancestral recombination graphs.** Shown are parameter choices used in the parsimony score at differing stages of genealogical inference by kwarg. At a given stage in the calculation, the set of recombination, mutation, and coalescence events inferred by previous steps determines a set of remaining sequences referred to as the *configuration*. Each row in the table represents parameter values used to calculate the parsimony score when evaluating move choices for the *next* step, at the indicated configuration complexity. 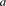 The maximum number of segregating sites remaining after any proposed move. 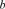 The number of recombination events in the proposed event (zero or one). 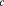 The resulting length of ancestral material after the proposed step (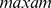 is the maximum across all proposed moves). 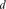 The number of sequences remaining after the proposed step (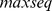 is the maximum across all proposed moves). 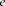 The Hudson-Kaplan lower bound on the remaining data after the proposed step. 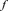 A composite lower bound combining the exact minimum across disjoint intervals. 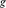 The haplotype lower bound. 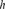 The exact minimum number of recombination events remaining. Parameters for these bounds are described in [15].(PDF)
