# Supplementary material for: Genealogy-Based Methods for Inference of Historical Recombination and Gene Flow and Their Application in Saccharomyces cerevisiae
Source: PLoS One. 2012 Nov 30;7(11):e46947. doi: 10.1371/journal.pone.0046947 (PMC3511476; doi:10.1371/journal.pone.0046947)
Supplement: Text S1 — Description of kwarg. (PDF) [file pone.0046947.s001.pdf]

Text S1: Description of `kwarg`

The software `kwarg` is a reimplementation of the algorithm presented in [1], which reconstructs ancestral recombination graphs (ARGs) backwards in time under the infinite-sites assumption. At each step, it (i) posits either a coalescence, mutation, or recombination event for the next event back in time; (ii) estimates which of these competing choices is the most parsimonious; and (iii) selects randomly from amongst the most parsimonious possible choices of event. Thus, we obtain a path of coalescence, mutation, and recombination events going backwards in time which correspond to an ARG. While [1] originally implemented a branch and bound algorithm for exploring all possible most-parsimonious paths, the method of `kwarg` only approximates this by computing a "parsimony score" to estimate the further number of moves required to complete the ARG subsequent to the move proposed. This score serves as a way to estimate the efficiency of each competing move, based upon specific features of the configuration resulting from the move. It is therefore not guaranteed to find an evolutionary history with a minimal number of recombination events, but with a suitable choice of parameters its reconstructions are in practice near-minimal. Furthermore, this approach runs with such efficiency that it is feasible to reconstruct many ARGs for every gene in the entire *S. cerevisiae* genome.

The parsimony score utilized by `kwarg` is customizable, and the precise implementation we chose is given in Table S3. The main contribution to the score is an estimate of the minimum number of remaining recombination events required to complete an evolutionary history for the data. To optimize the tradeoff between speed and efficiency, we used a combination of the Hudson-Kaplan lower bound (hk) [2], the haplotype bound (hb) [3], a composite of exact bounds over short stretches of sequence ($eagl$) [1], and the exact minimum number of recombination events over the whole sequence ($rmin$) [1], where larger datasets would rely on quicker and less sharp bounds and smaller datasets would rely on slower but sharper bounds. Briefly, in the syntax of `kwarg`, the choice of bound depends on the complexity of the configuration under examination. Configuration complexity was measured by the variables $maxlen$ and $maxam$, the maximum number of segregating sites and maximum length of ancestral material, respectively, remaining across all the proposed moves. The length of ancestral material for a proposed move, am, is defined to be the sum across sequences in the current configuration of the number of sites which are ancestral to a site in the original present-day sample of sequences. So for example, suppose we are at a stage where we have to choose between all possible moves from a configuration such that, after any proposed move there are at most fifty remaining segregating sites, and the total length of ancestral material is less than one hundred. Then, according to the fourth row of Table S3, the score on which the next proposed move is based is the sum of: the number of recombination events involved in the proposed move (r), its effect on reducing the total amount of ancestral material amongst all possible moves ($am/maxam$), its effect on reducing the total number of sequences remaining in the dataset amongst all possible moves ($seq/maxseq$), and the *exact* minimum number of recombination events remaining in the dataset after the move has been carried out ($rmin$). If the reconstruction of any ARG required more than two hours of computing time, then we switched to a slightly faster scoring scheme for that gene (Table S3, bottom four rows). The bounds $eagl$ and hb take arguments which fine-tune the speed and accuracy with which they are computed. Further details on these and the scoring function can be found in the documentation for `kwarg` [1].

References

1. Lyngsø RB, Song YS, Hein J (2005) Minimum recombination histories by branch and bound. In: Proc. of 2005 Workshop on Algorithms in Bioinformatics. Berlin, Germany: Springer-Verlag LNCS, pp. 239-250. The `kwarg` software package is available from http://www.stats.ox.ac.uk/∼lyngsoe/section26/.

2. Hudson RR, Kaplan NL (1985) Statistical properties of the number of recombination events in the history of a sample of DNA sequences. Genetics 111: 147–164.

3. Myers SR, Griffiths RC (2003) Bounds on the minimum number of recombination events in a sample history. Genetics 163: 375–394.
