# Supplementary figures and images for: Genealogy-Based Methods for Inference of Historical Recombination and Gene Flow and Their Application in Saccharomyces cerevisiae
Source: PLoS One. 2012 Nov 30;7(11):e46947. doi: 10.1371/journal.pone.0046947 (PMC3511476; doi:10.1371/journal.pone.0046947)

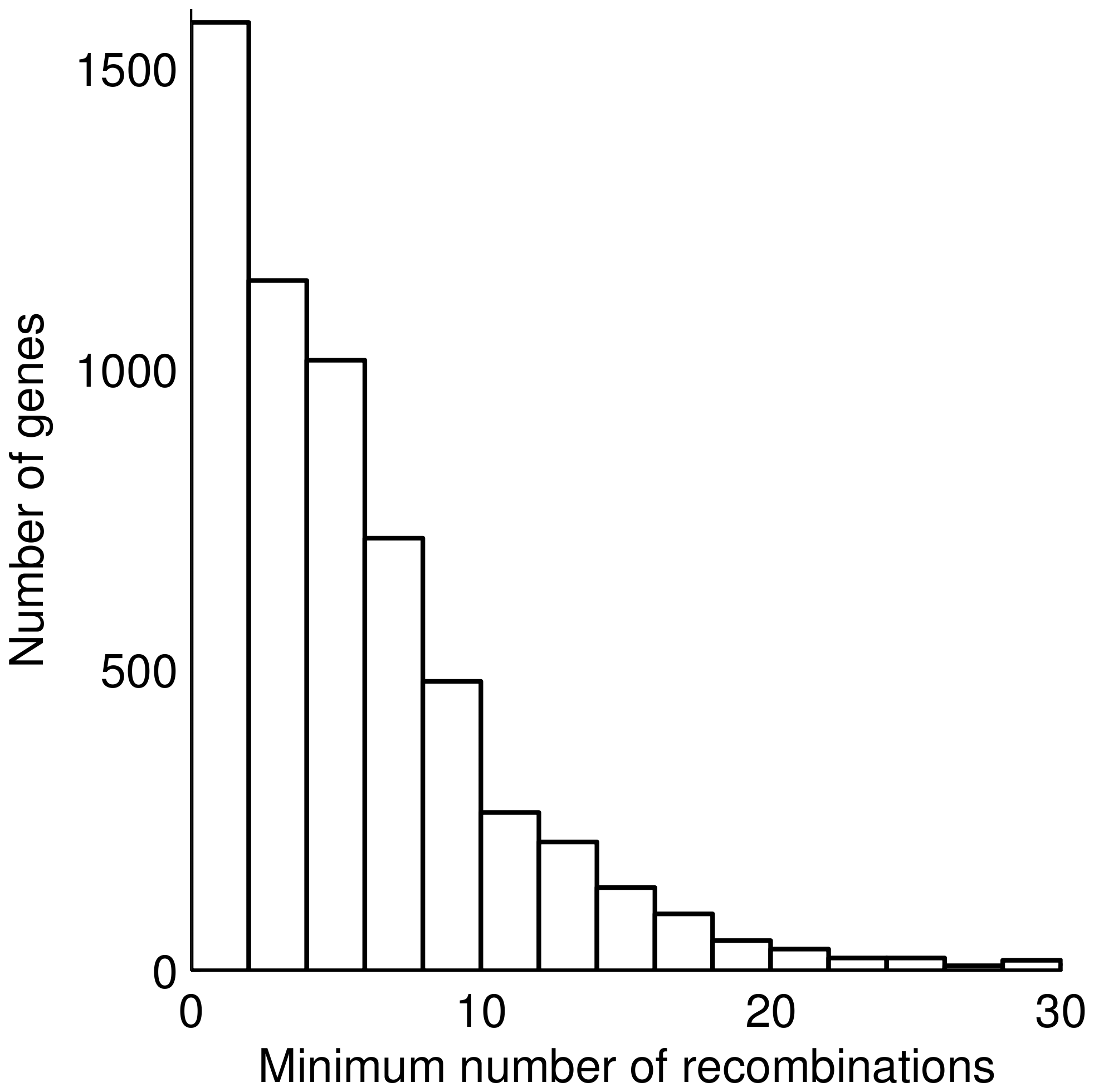

Supplement: Figure S1 — Distribution of the number of inferred recombinations across yeast genes. Shown is the inferred minimum number of recombination events per gene from ancestral recombination graphs for 5842 yeast genes; genes with five or fewer SNPs and those for which independent ancestral reconstructions did not agree on the number of recombination events were eliminated from the data set. (TIF) [file pone.0046947.s003.tif]
