# Supplementary material for: Genealogy-Based Methods for Inference of Historical Recombination and Gene Flow and Their Application in Saccharomyces cerevisiae
Source: PLoS One. 2012 Nov 30;7(11):e46947. doi: 10.1371/journal.pone.0046947 (PMC3511476; doi:10.1371/journal.pone.0046947)
Supplement: Table S1 — Genome-wide estimates of the migration parameter under a symmetric island model, as inferred from . Each row reports estimated population differentiation and migration parameter values for one pair of yeast populations. In a given gene window, is calculated as one minus the ratio of within- to between-population diversity, as given by the mean number of pairwise sequence differences. Mean is the mean of this value across genes, weighted by length. A point estimate of the migration parameter in a symmetric five-island model, inferred from mean and using a theoretical prediction of the relationship between and (see Materials & Methods). Also given are percentiles for this estimate, inferred from the distribution of across genes. (PDF) [file pone.0046947.s004.pdf]

Table S1. Genome-wide estimates of the migration parameter M under a symmetric island model, as inferred from F_{ST}.

Population pair	Mean F_{ST} [a]	$\widehat{M}^b$	(5, 95)th percentiles for $\widehat{M}$
M vs. N.A.	0.96	0.07	(0, 0.48)
M vs. S	0.84	0.31	(0, 1.52)
M vs. W.A.	0.97	0.05	(0, 0.32)
M vs. W/E	0.93	0.13	(0, 0.57)
N.A. vs. S	0.69	0.70	(0, ∞)
N.A. vs. W.A.	0.98	0.04	(0, 0.13)
N.A. vs. W/E	0.90	0.18	(0, 0.93)
S vs. W.A.	0.79	0.41	(0, 2.40)
S vs. W/E	0.82	0.35	(0, 1.45)
W.A. vs. W/E	0.91	0.15	(0, 0.64)

Each row reports estimated population differentiation and migration parameter values for one pair of yeast populations.

[a] In a given gene window, F_{ST} is calculated as one minus the ratio of within- to between-population diversity, as given by the mean number of pairwise sequence differences. Mean F_{ST} is the mean of this value across genes, weighted by length.

[b] A point estimate of the migration parameter M in a symmetric five-island model, inferred from mean F_{ST} and using a theoretical prediction of the relationship between M and F_{ST} (see Materials & Methods). Also given are percentiles for this estimate, inferred from the distribution of F_{ST} across genes.
