# Supplementary material for: Genealogy-Based Methods for Inference of Historical Recombination and Gene Flow and Their Application in Saccharomyces cerevisiae
Source: PLoS One. 2012 Nov 30;7(11):e46947. doi: 10.1371/journal.pone.0046947 (PMC3511476; doi:10.1371/journal.pone.0046947)
Supplement: Table S2 — Changes in caused by deviations from the symmetric five-island model. Each cell reports an estimate, by simulation, of , where is the fraction of disrupting recombination events toggling the outgroup status of population A which also occur in a lineage ancestral to population A, under the indicated model; is the analogous quantity for a five-island population model with constant, symmetric migration with parameter and reference effective population size per population, which reverts to a fully panmictic population generations ago. Under this model, . A. One population has a different effective population size. The first column perturbs the population size of the population experiencing the disrupting recombination event, denoted A, while the second column perturbs the population size of one of the other four populations, denoted B. B. The population whose outgroup status is disrupted, A, experiences higher incoming (first column) or outgoing (second column) migration rates, relative to the other populations. C. An alternative model in which certain populations have an older origin. In the first column, each island joins a single, panmictic, population generations ago, with the exception of population A, which joins the panmictic population at the time indicated. In the second column, it is instead one of the other four populations, which we call B, that joins the ancestral panmictic population farther back in time. (PDF) [file pone.0046947.s005.pdf]

Table S2. Changes in f_A caused by deviations from the symmetric five-island model.

A.

Population size	Population A	Population B
$0.2N$	0.495	1.101
N	1	1
$5N$	2.014	0.819

B.

Relative migration rate	Into A	Out of A
5	0.906	1.380
10	0.875	1.523
50	1.087	1.328

C.

Population age (generations)	Population A	Population B
$6N$	1.101	1.101
$9N$	0.958	1.160
$12N$	1.195	1.122

Each cell reports an estimate, by simulation, of f_A/f_{A0}, where f_A is the fraction of disrupting recombination events toggling the outgroup status of population A which also occur in a lineage ancestral to population A, under the indicated model; f_{A0} is the analogous quantity for a five-island population model with constant, symmetric migration with parameter $M = 0.1$ and reference effective population size N per population, which reverts to a fully panmictic population $12N$ generations ago. Under this model, $f_{A0} = 0.287$.

A. One population has a different effective population size. The first column perturbs the population size of the population experiencing the disrupting recombination event, denoted A, while the second column perturbs the population size of one of the other four populations, denoted B.

B. The population whose outgroup status is disrupted, A, experiences higher incoming (first column) or outgoing (second column) migration rates, relative to the other populations.

C. An alternative model in which certain populations have an older origin. In the first column, each island joins a single, panmictic, population $6N$ generations ago, with the exception of population A, which joins the panmictic population at the time indicated. In the second column, it is instead one of the other four populations, which we call B, that joins the ancestral panmictic population farther back in time.
