# Supplementary material for: Genealogy-Based Methods for Inference of Historical Recombination and Gene Flow and Their Application in Saccharomyces cerevisiae
Source: PLoS One. 2012 Nov 30;7(11):e46947. doi: 10.1371/journal.pone.0046947 (PMC3511476; doi:10.1371/journal.pone.0046947)
Supplement: Table S3 — Parameters for parsimony score used in kwarg to reconstruct ancestral recombination graphs. Shown are parameter choices used in the parsimony score at differing stages of genealogical inference by kwarg. At a given stage in the calculation, the set of recombination, mutation, and coalescence events inferred by previous steps determines a set of remaining sequences referred to as the configuration. Each row in the table represents parameter values used to calculate the parsimony score when evaluating move choices for the next step, at the indicated configuration complexity. The maximum number of segregating sites remaining after any proposed move. The number of recombination events in the proposed event (zero or one). The resulting length of ancestral material after the proposed step ( is the maximum across all proposed moves). The number of sequences remaining after the proposed step ( is the maximum across all proposed moves). The Hudson-Kaplan lower bound on the remaining data after the proposed step. A composite lower bound combining the exact minimum across disjoint intervals. The haplotype lower bound. The exact minimum number of recombination events remaining. Parameters for these bounds are described in [15]. (PDF) [file pone.0046947.s006.pdf]

Table S3. Parameters for parsimony score used in `kwarg` to reconstruct ancestral recombination graphs

Runtime per ARG	Configuration complexity	Parsimony score $=$ $r^b + \max(am^c/maxam, seq^d/maxseq) +$ the following:
< 2 hrs	$maxlen^a > 100$	$2 \times hk^e$
	$50 < maxlen \leq 100$	$1.5 \times \max(eagl(7)^f, hb(2,50,6)^g)$
	$maxlen \leq 50, maxam \geq 100$	$1.25 \times \max(eagl(10), hb(2,-1,-1))$
	$maxlen \leq 50, maxam < 100$	$rmin^h$
> 2 hrs	$maxlen > 100$	$2 \times hk$
	$50 < maxlen \leq 100$	$1.5 \times \max(eagl(5), hb(2,50,6))$
	$maxlen \leq 50, maxam \geq 25$	$1.25 \times \max(eagl(7), hb(2,50,10))$
	$maxlen \leq 50, maxam < 25$	$rmin$

Shown are parameter choices used in the parsimony score at differing stages of genealogical inference by `kwarg`. At a given stage in the calculation, the set of recombination, mutation, and coalescence events inferred by previous steps determines a set of remaining sequences referred to as the *configuration.* Each row in the table represents parameter values used to calculate the parsimony score when evaluating move choices for the *next* step, at the indicated configuration complexity.

[a] The maximum number of segregating sites remaining after any proposed move.

[b] The number of recombination events in the proposed event (zero or one).

[c] The resulting length of ancestral material after the proposed step (*maxam* is the maximum across all proposed moves).

[d] The number of sequences remaining after the proposed step (*maxseq* is the maximum across all proposed moves).

[e] The Hudson-Kaplan lower bound on the remaining data after the proposed step.

[f] A composite lower bound combining the exact minimum across disjoint intervals.

[g] The haplotype lower bound.

[h] The exact minimum number of recombination events remaining.

Parameters for these bounds are described in Lyngsø *et al.* (2005).
